# Diffusion-Weighted MRI and Quantitative Biophysical Modeling of Hippocampal Neurite Loss in Chronic Stress

**DOI:** 10.1371/journal.pone.0020653

**Published:** 2011-07-01

**Authors:** Peter Vestergaard-Poulsen, Gregers Wegener, Brian Hansen, Carsten R. Bjarkam, Stephen J. Blackband, Niels C. Nielsen, Sune N. Jespersen

**Affiliations:** 1 Center for Functionally Integrative Neuroscience, Aarhus University, Aarhus, Denmark; 2 Center for Basic Psychiatric Research, Aarhus Psychiatric University Hospital, Risskov, Denmark; 3 Center for Insoluble Protein Structures and Interdisplinary Nanoscience Center, Department of Chemistry, Aarhus University, Aarhus, Denmark; 4 Department of Neuroscience, McKnight Brain Institute and The National High Magnetic Field Laboratory, University of Florida, Gainesville, Florida, United States of America; 5 Department of Neurosurgery, Aarhus University Hospital, Aarhus, Denmark; City of Hope National Medical Center and Beckman Research Institute, United States of America

## Abstract

Chronic stress has detrimental effects on physiology, learning and memory and is involved in the development of anxiety and depressive disorders. Besides changes in synaptic formation and neurogenesis, chronic stress also induces dendritic remodeling in the hippocampus, amygdala and the prefrontal cortex. Investigations of dendritic remodeling during development and treatment of stress are currently limited by the invasive nature of histological and stereological methods. Here we show that high field diffusion-weighted MRI combined with quantitative biophysical modeling of the hippocampal dendritic loss in 21 day restraint stressed rats highly correlates with former histological findings. Our study strongly indicates that diffusion-weighted MRI is sensitive to regional dendritic loss and thus a promising candidate for non-invasive studies of dendritic plasticity in chronic stress and stress-related disorders.

## Introduction

The adverse effects of chronic stress on physiology, learning and memory are well described [1] and are known to be involved in the development of anxiety disorders such as post-traumatic stress disorder (PTSD) and major depressive illness [2]. The stress response of the body acts via a glucocorticoid-mediated negative feedback on the hypothalamus-pituitary-adrenal axis upon which the hippocampus has a major regulatory role [3]. Being of central importance in spatial learning and memory, the hippocampus is a highly vulnerable brain structure susceptible to the damaging effects of chronic stress and circulating adrenal steroids [4].

Animal studies of increased glucocorticoid levels induced either by stressful environments or by exogenous administration have identified three major effects on neural plasticity in the hippocampus: First, modified intrinsic excitability and activity-dependent synaptic plasticity have been reported in several studies. For instance, a distinct reduction in long-term potentiation (LTP) coupled with increased long-term depression (LTD) in the CA1 pyramidal cell region was found in rats exposed to even mild levels of stress [5]. Second, high levels of glucocorticoids or stress can result in inhibition or cessation of neurogenesis in the dentate gyrus (DG) of the hippocampus [6], [7]. Third, there is profound evidence that chronic high level stress or glucocorticoid administration in rats and primates is associated with loss of apical dendritic material of pyramidal neurons and even neuronal death [8], [9], especially in CA3 [9], [10], but also in the CA1 region [11]. In moderate durations of stress or glucocorticoid administration, this effect has been reported in CA3 pyramidal neurons and correlates with a reduced performance in both short-term spatial and other types of memory tasks in rats [12]–[18]

These studies used morphometric parameters such as dendritic length and branching numbers of neurons visualized by histology [4]. Hence, further in vivo investigation of dendritic remodeling during development, monitoring or treatment of stress and stress-related diseases such as depression and PTSD is limited by the absence of a sensitive and non-invasive neuroimaging method.

Magnetic Resonance Imaging (MRI) sensitized to water self diffusion (DWI) has proven to be uniquely sensitive to subtle changes in brain tissue microstructure in a number of reports [19], [20], notably early *in vivo* detection of regional cerebral ischemia [21]. The intrinsic water diffusion anisotropy of white matter can be measured *in vivo* by diffusion tensor MR imaging (DTI) and used for *in vivo* axonal fiber tracking in the brain [22], thus proving useful in studies of a number of white matter diseases [23]. Diffusion anisotropy derived from the diffusion tensor model – which simply models the water diffusion in each voxel as an ellipsoid - has been used to explore the effects of stress on white matter development. For instance, in monkeys exposed to intermittent social separation stress, it was found that the ventromedial prefrontal white matter diffusion anisotropy was greater than in controls [19]. Early life stress was associated with reduced fractional diffusion anisotropy in the anterior internal capsule in monkeys [24]. In humans, children with PTSD were shown to have reduced diffusion anisotropy in the medial and posterior corpus callosum which may be attributed to reduced myelination or changes in axonal structure [20].

In gray matter, cortical diffusion anisotropy has been shown to be sensitive to the level of neuronal migration in developing ferret brains [25]. While having an extreme sensitivity to changes occurring in the underlying tissue microstructure, the parameters that describe the diffusion-weighted signal, such as the diffusion coefficient or the diffusion anisotropy, suffer from a lack of specificity to the microstructural geometry of neuronal tissue [23].

Many attempts have been made to develop a biophysical model of the diffusion-weighted signal capable of quantifying the microstructure of the neuronal system in terms of physically interpretable parameters [26]–[28]. A promising biophysical model of both gray and white matter brain tissue [29] was recently validated towards both quantitative light- and electron microscopy through demonstrating a very strong correlation with the neurite density obtained from DWI in several brain regions [30]. We hypothesize that appropriate DWI and this specific type of biophysical modeling of neuronal tissue may offer the ability to detect and quantify the underlying regional dendritic remodeling observed in standardized studies of chronic stress. In this study, a validated model of neurite density [30] of high field DWI data were used to detect the regional microstructural changes that occur in the rat hippocampus after a 21 day period of standardized chronic stress.

## Materials and Methods

### Animals and Stress paradigm

Ten adult male Wistar rats aged 9-10 weeks (300 grams, Taconic MB, Denmark) were randomly and evenly divided into a group receiving exposure to stress and a control group. All animal experiments were approved in accordance with all guidelines and regulations of The Danish National Committee for Ethics in Animal Experimentation (Approved by the Local Erthics comittee for Aarhus County, Denmark, authorization number 2007/561-1378). The animals were housed in groups of two with *ad libitum* access to food and water. All animals were maintained in a temperature controlled room, with a light/dark cycle of 12/12 hours (lights on at 06.00 a.m.). During the three week stress period, the control rats remained in their home cages with daily handling, i.e. each rat was taken out of the cage, gently handled for approximately 20 seconds, and subsequently returned to the home cage. In a separate room, the stress group was subjected to a 6 hour daily restraint stress schedule (09.00 a.m. to 3.00 p.m.) for 21 days in transparent acrylic restrainers secured at the head and tail, with an intensive light source above (1000 Lux).

### Tissue preparation

Animals were euthanized by an injection of 5 ml Sodium-pentobarbital (20 mg/ml). When deep reflexes were no longer present, the animals were exsanguinated during intraaortic perfusion with isotonic saline containing heparin (10 IU/mL), followed by perfusion-fixation using 4% paraformaldehyde dissolved in phosphate-buffered saline (pH 7.4). The brains were removed and immersion-fixed in a fresh 4% formaldehyde solution at room temperature and stored for two weeks. The brains were bisected mid-sagittally, and the right hippocampi removed by gross dissection using the lateral ventricle as a reference [31]. The samples were then immersion-fixed in fresh 4% formaldehyde solution until DWI data collection.

### DWI data collection

Prior to MRI, the brains were washed for 48 hours in a phosphate buffered saline solution (pH = 7.4) in order to remove formalin and reduce signal loss [32]. Each specimen was then placed in a thin-walled, standard 5 mm diameter NMR glass tube with the longitudinal direction of the excised hippocampus parallel to the tube axis, and positioned in a 16.4 T (700 MHz for ^1^H) vertical, wide bore Bruker Avance II NMR spectrometer (Bruker BioSpin GmbH, Rheinstetten, Germany), equipped with a gradient system capable of up to 300 Gauss/cm. All experiments were performed at a controlled temperature of 21°C.

A standard spin echo Stejskal-Tanner diffusion-weighted sequence was used to acquire a total of 54 diffusion directions chosen from a 54 point spherical 9-design [33]. A total of 9 shells (b-values = 0, 2000, 3000, 4000, 5000, 6000, 8000, 10000, 15000 s/mm^2^) were acquired with 6 unique directions on each shell. The remaining diffusion and imaging parameters were as follows: TR = 2.5 s, TE = 14.3 ms, data matrix = 64×64, field of view  = 4.5 mm ×4.5 mm, axial slice thickness  = 0.32 mm, and Δ/δ  = 8/2 ms. Four averages were acquired per direction. Total acquisition time was 9 h 36 min.

### Biophysical model of water-self diffusion in neuronal tissue

The model is based on a biophysical description of brain microstructure [29] with the fundamental assumption that water diffusion can be described in terms of two non-exchanging components. One component is associated with diffusion in cylindrically symmetric structures, such as dendrites and axons (collectively called neurites) with exchange of water being sufficiently slow to be considered impermeable on the time scale of the diffusion imaging experiment. The net signal from this component then arises as a sum of the signal from all neurites weighted by an orientation distribution function, i.e. a probability density function specifying the number of neurites in every direction. The second component of the diffusion signal accounts for diffusion everywhere else, in particular in cell bodies, extracellular space, and glia cells. Here, diffusion is hindered and approximated by Gaussian isotropic diffusion with an effective diffusion constant. Several cytoarchitectural parameters can be extracted from this framework [30]. Here, we estimate the voxel-wise neurite density from the volume fraction of the net signals from modeled neurites. The voxel-wise mean diffusivity of the extra-cylindrical space was estimated for use only in defining the hippocampal subregions (see next section).

### Analysis and statistics

An experienced neuroanatomist (CRB), blinded to which group the rat belonged, defined regions of interest (ROI) in the stratum oriens, pyramidal cell layer, stratum radiatum and the stratum lacunosum moleculare of the central CA1 and CA3 regions, as well as the molecular and the granule cell layer of the DG. The definition of the ROI was performed on the mean diffusivity maps with very high signal in cell layers at a position of the rostral-caudal axis of approximately –2.9 mm relative to bregma [34]. Two data sets were discarded, one from each group. One data set contained a tear in the hippocampus at the position of the rostral-caudal axis where we applied the analysis, and the other data set contained image artifacts of unknown origin in a portion of the diffusion directions. Despite efforts to perform an analysis without these directions, a reliable analysis was not obtained.

A Wilcoxon rank sum test was applied to the group mean of all pixels in the defined ROIs obtained from the modeled neurite density maps. Differences were considered significant at a level of P<0.05 (uncorrected for multiple comparisons between the different regions). The relative difference of the modeled neurite density of the groups was compared to formsimilar literature light microscopy studies of the rat hippocampus after 21 days of standardized chronic stress.

## Results

**Figure 1:**
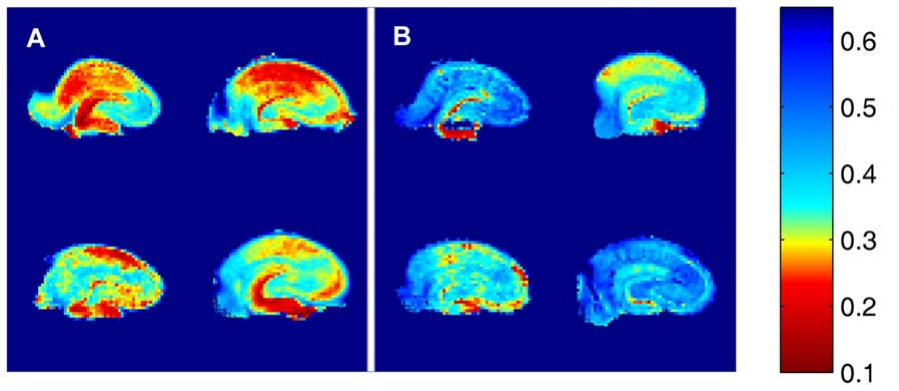
Neurite density maps of stressed and control rat hippocampi. (**A**) stressed rats, (**B**) control rats. The color bar shows the normalized neurite density. Note: the highest red intensity on the color bar refers to lowest neurite density.

**Figure 2:**
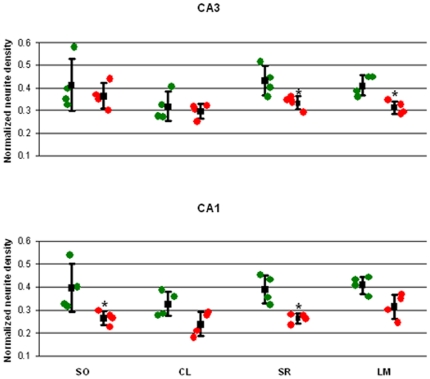
CA3 and CA1 normalized neurite density of stratum oriens (SO), cell layer (CL), stratum radiatum (SR) and stratum lacunosum moleculare layer (LM). Individual data from controls rats (green dots) and stressed rats (red) are shown in conjunction with the mean values and standard deviation (black). ^*^P<0.05.

The modeled neurite density of the apical CA3 and CA1 regions was lower in the stressed animals than in the controls as can be seen on maps of the hippocampal normalized neurite density (Figure 1A,B). The modeled neurite density in both the CA3 stratum radiatum and the stratum lacunosum moleculare of the stressed group were significantly lower than in the controls (Table 1, Figure 2). A similar reduction was seen in the CA1 (Table 1, Figure 2). However, no statistical difference in modeled neurite density of the pyramidal cell layers in both CA3 and CA1 could be detected between groups (Table 1, Figure 2). Statistical difference in modeled neurite density of the stratum oriens layer could be detected between groups in CA1 but not CA3 (Table 1, Figure 2).

**Table 1:** Normalized hippocampal modeled neurite density in stressed and control groups of rats.

Region	Subregion	Stressed	Control	P-value
CA1	SO	0.27±0.03*	0.40±0.10	0.03
	CL	0.24±0.05	0.33±0.05	0.11
	SR	0.26±0.02*	0.39±0.06	0.03
	LM	0.31±0.05	0.41±0.04	0.06
CA3	SO	0.36±0.06	0.41±0.12	0.69
	CL	0.30±0.03	0.32±0.06	0.69
	SR	0.33±0.03*	0.43±0.07	0.03
	LM	0.31±0.03*	0.41+0.05	0.03
DG	GL	0.23±0.005*	0.31±0.04	0.03
	ML	0.28±0.03*	0.36±0.03	0.03

All values are mean ± standard deviation of the subregion (n = 4 for both groups). Subregion abbreviations are: stratum oriens (SO), pyramidal cell layer (CL), stratum radiatum (SR), stratum lacunosum moleculare layer (LM), granule cell layer (GL), molecular layer (ML).

*Significant difference between stressed and control group (Wilcoxon rank sum test P<0.05 uncorrected for multiple comparisons).

**Figure 3:**
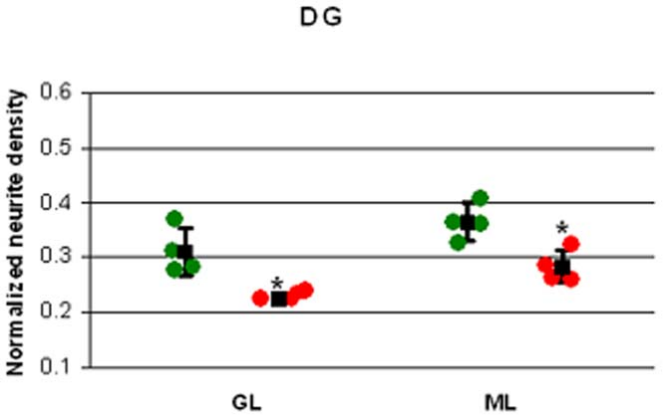
DG normalized neurite density of granule cell layer (GL) and molecular layer (ML). Individual data from controls rats (green dots) and stressed rats (red) are shown in conjunction with the mean values and standard deviation (black). ^*^P<0.05.

The modeled neurite density in the DG granule cell layer of the stressed rats was lower than in the controls (Table 1, Figure 1 and 3). The DG molecular layer neurite density of the stressed group was likewise significantly lower than in the controls (Table 1, Figure 1 and 3). Interestingly, the variance of the modeled neurite density in the stressed group, in regions where there were larger differences from the control group, was generally lower than the control group. This is seen especially in the granule and molecular layers of the DG (see Figure 3).

## Discussion

In a 21 day restraint-stressed rat model we found significantly lower DWI measured neurite density in the hippocampal CA3 apical areas compared to controls. No differences were found in the stratum oriens region or the pyramidal cell layer.

Our findings in CA3 are consistent with earlier histological studies in rats exposed to similar 6 hour/21 day restraint stress models. These studies all showed that while the total dendritic length of the basal dendrites in stratum oriens did not change, the apical dendrites of these neurons were reduced by approximately 18 to 36% [13], [15]–[17], [35]–[38]; an average reduction of 27%.

The general histological findings of a reduced dendritic length confined to only the apical part of hippocampal pyramidal neurons due to stress were also reflected in a reduced neurite density estimated by DWI in our study. We note that the dendritic length cannot be directly compared to the neurite density (which is the volume fraction of dendrites and axons in the biophysical model employed) obtained in this study. However, under the reasonable assumption that the dendritic length correlates quasi-linearly to the volume fraction of dendrites in the dendritic tree, our reported 24% average reduction of apical dendrite density in the CA3 region is strikingly similar to the findings of former histological studies which found an average dendritic length reduction of 27% when employing similar 21 day restraint stressed models in rats.

Previous 21 day restraint stressed rat studies did not include the CA1 region [13],[15]–[17],[35],[37],[39] or found no changes in the length of the apical dendritic tree CA1 [38]. We have found a reduction in CA1 modeled neurite density in the stratum radiatum of 33%. A more recent study supports CA1 dendritic retraction by demonstrating a 33% decrease of the terminal segment length of the dendritic tree in CA1 and a 25% reduction of the total dendritic length in the CA3 region following 30 days restraint stress [11]. Also, only 6 days of activity stress in rats caused a 33% reduction of the total dendritic length in CA1 apical dendrites [40].

Subtle differences in the restraint stress induction models may affect the stress response considerably and could be part of the explanation for these apparent differences [41]. Further, despite being invented a century ago, the mechanisms that are responsible for Golgi-Cox staining have not yet been completely elucidated suggesting that differences in fixation procedures might affect the staining of neurons and thereby the measurement of total dendritic length and branching numbers [42].

Future studies should include both histology and DWI on the same specimens to more directly compare the specificity of these methods in the estimation of regional neurite density. This would also shed more light on the potential confounds from the possible sensitivity of DWI to other structural differences than dendritic retraction between groups of stressed and control animals. Its known that i.e. the apical, but also the basal dendritic spine density in CA1 are increased due to stress induction [43] which might affect water diffusion dynamics in addition to the changed water diffusion dynamics due to dendritic retraction. Also changes in the dendritic spine density of the CA3 (the socalled thorny excrecences) shown in induced stress could principly have an effect on the DWI. It is still controversial in the histolgy literature whether this is the case in stress [11], [44]. A close relationship between neurite structure and hippocampus-dependent learning and memory using a water maze has been demonstrated in rats [11], an indication that such measures should be included in future correlative studies of DWI and histological and stereological analysis of brains from stressed animals.

There was a decreased - but not significant (P<0.06) - of modeled neurite density in the DG molecular layer of 25% in our 21 day study, while an earlier study found a 38% reduction of total dendritic length in a 30 day restraint stress rat model [11].

In the DG granule layer of the the stressed group a 26% reduction of the modeled neurite density was found in this study. However, a reduction of the estimated normalized neurite density within the employed model of diffusion must be interpreted as a reduced volume fraction of cylindrical segments (dendrites or processes) and thus, an increased volume fraction of the second compartment. In the cell layer, this second compartment is the sum of the extracellular space and the cell somas: both of which exhibit hindered, but approximately Gaussian isotropic diffusion. Thus, this model which was developed to estimate neurite density cannot distinguish between these two subcompartments in the cell layers, and we conclude only that the total volume fraction of Gaussian isotropic diffusion is increased after 21 day stress.

Nevertheless, the finding that DWI detects a change in the water diffusion properties in the cell layers of the DG is highly interesting because it indicates sensitivity to the structural changes associated with neurogenesis. Earlier reports using corticosterone injections of rats or psycho-social stress (in tree shrews) have demonstrated a reduced rate of progenitor cell birth in the hilus [6], [45]. Progenitor cells have been suggested to migrate into the granule cell layer within a 21 day period [45]. This could indicate that there is a decrease of cellular density in the granule layer causing the extracellular space to expand which could be the basis for the higher volume fraction of the Gaussian diffusion observed here. As no changes have been detected in the pyramidal cell layers of CA1/CA3 in this study, matching the general findings of no neuronal loss in 21-day stress studies, this suggests that DWI is a marker of structural changes in the cellular region, although not a specific one. Further research on biophysical models that include the cellular layer is needed to further explore the specificity to the structural changes in cell layers.

Chronic stress selectively reduces the total hippocampal volume by 3% in rats after a 21 day restraint stress period [46]. It is currently not known how regional shrinkages generate a reduction of the total hippocampal volume or how the intra- and extra-cellular fractions are modulated as a result of dendritic retraction. A combination of mapping the regional neurite density by DWI in the entire hippocampus and total hippocampal volume estimation might explain this and should be included in further investigations.

In this *in vitro* study of a standard restraint stressed rat model, we have demonstrated the sensitivity of DWI to underlying microstructural changes, probably to a loss in dendritic material, that agree very well with former histological findings. We predict that a number of stress research fields can benefit from an imaging method that is sensitive to changes in dendritic structure. First, studies of how the type of stressor, context, duration, gender, age and genes are related to neurite structure would be more feasible with a non-invasive neuroimaging technique. Next, the reversibility of chronic stress has been the subject of many discussions [2]. Most studies show that the dendritic retraction in the hippocampus after a 21 day period of restraint stress seems to be reversible within approximately 10 days [36], [47]. However, brain structures involved in the control of the HPA-axis are interconnected, so the prefrontal cortex and the hippocampus are both affected by remodeling of neurons in the basolateral complex of the amygdala (BLA) which is an important substrate in integrating the influence of hormonal and neurotransmitter systems on memory consolidation [2]. Interestingly, and in contrast to the effects on the hippocampus, chronic stress produces dendritic growth in the BLA that does not recover even after a longer period in stress-free environments [2]. The continued hypertrophy of neurons in the BLA was also reflected in a persistent state of heightened anxiety and thus may be a consequence of a cellular substrate promoting high anxiety levels. An *in vivo* neuroimaging method sensitive to dendritic remodeling would be an important tool to understand these mechanisms and develop treatment of anxiety disorders like PTSD, but would also be applicable to numerous other fields including development, aging, seizures, ischemia, rehabilitation and depression treatment.

However, there are a number of technical challenges to be addressed before this method can be applied *in vivo*. First, much shorter examination times are required. This implies that the DWI acquisition must be simplified (i.e. reducing the number of b-values, directions or averages) in a manner that does not preclude reliable estimation of biophysical model parameters[48]. Second, the lower field strengths (typically 5–11 Tesla for animal magnets, 1.5–3 Tesla for human magnets) and lower performance of the gradient systems in MRI systems for live animal and especially for human studies will result in limited signal-to-noise ratio and spatial resolution. The limited range of diffusion times and thereby echo times for DWI in clinical scanners compared to experimental high field systems with high performance gradient systems needs further attention due to the sensitivity to the characteristic diffusion length of water molecules and tissue subcompartmental T_2_ relaxation. Further studies, beginning with in vivo animal studies, are needed to explore the ramifications and possible solutions to these issues.

In summary, this study shows that there are strong indications that DWI is sensitive to the dendritic retraction of rat hippocampal neurons that undergo a 21 day restraint stress. The regional degree of hippocampal neuritic loss found by this method was in agreement to neuritic loss measured using light microscopy in earlier studies of 21 day restraint stress. Thus, DWI might support or even in some cases substitute histology in a number of *in vitro* applications as well as being the only present candidate for a non-invasive *in vivo* neuroimaging method – apparently sensitive to dendritic remodeling - in studies of anxiety disorders, depression, several neuro-degenerative disorders and development and aging of the brain. Further validation studies and technical developments are needed to fully elucidate this potential.
